# Supplementary material for: Redox-homogeneous, gel electrolyte-embedded high-mass-loading cathodes for high-energy lithium metal batteries
Source: Nat Commun. 2022 May 9;13:2541. doi: 10.1038/s41467-022-30112-1 (PMC9085813; doi:10.1038/s41467-022-30112-1)
Supplement: Supplementary file 1 — Supplementary information [file 41467_2022_30112_MOESM1_ESM.pdf]

Supplementary information

Redox-homogeneous, gel electrolyte-embedded high-mass-loading cathodes for high-energy lithium metal batteries

Jung-Hui Kim, Ju-Myung Kim, Seok-Kyu Cho, Nag-Young Kim, Sang-Young Lee✉

Supplementary figures

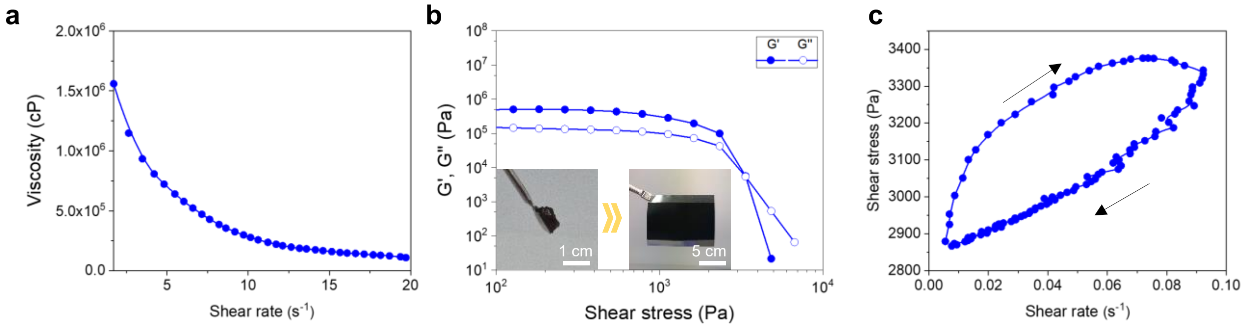

Supplementary Figure 1 | Rheological properties of the BNQS electrode paste. a Viscosity as a function of shear rate. **b** Viscoelastic properties (G′ and G″) as a function of shear stress (insets show photographs of the electrode paste and the printed paste on an Al current collector). **c** Rheogram showing the hysteresis loop.

a

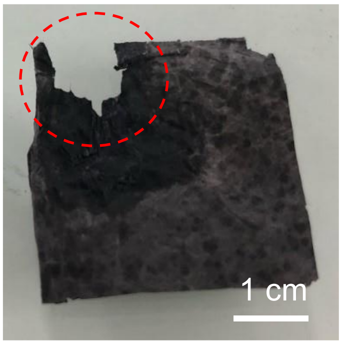

b

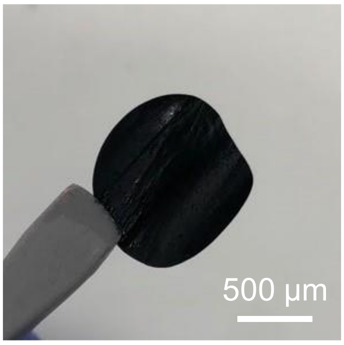

Supplementary Figure 2 | Structural stability of the electroconductive mat upon contact with electrode pastes. a Photograph of the SWCNT-wrapped PEI mat. **b** Photograph of the SWCNT-wrapped PEI-TPPTA mat.

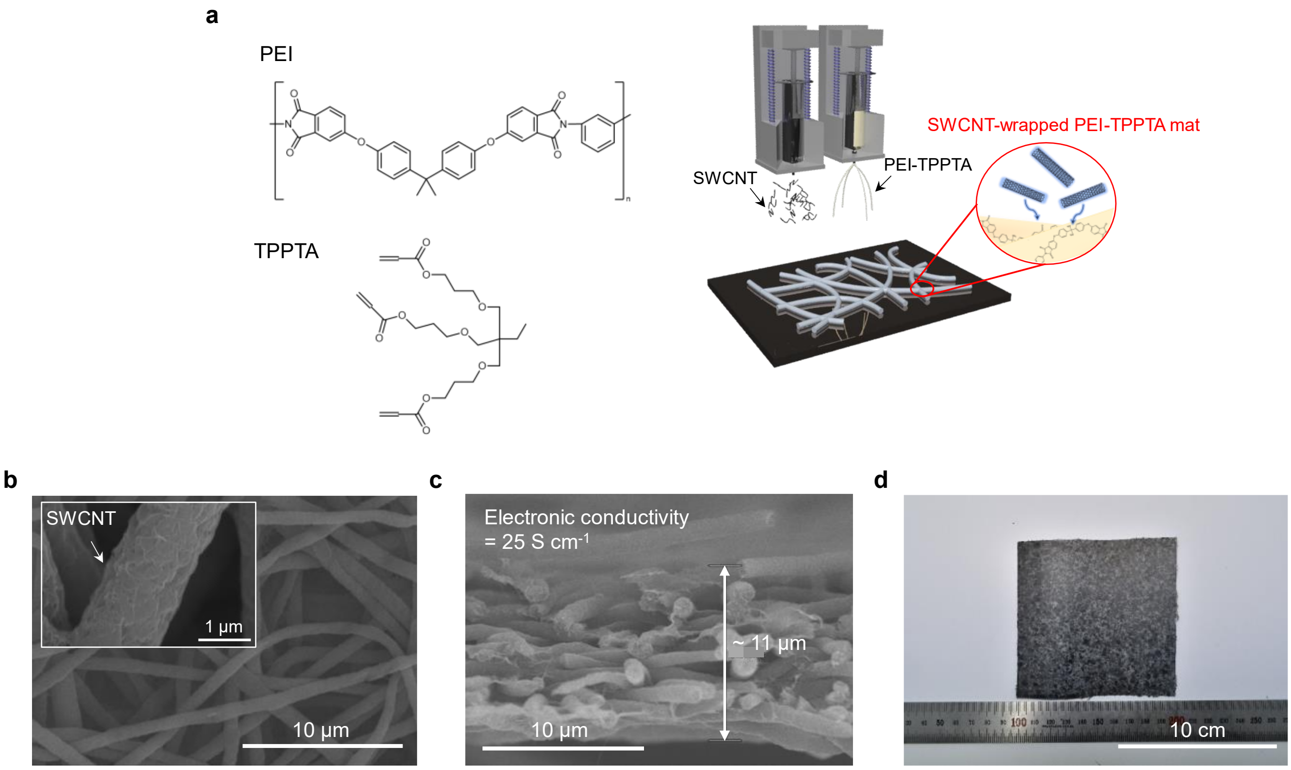

Supplementary Figure 3 | **a** Schematic illustration depicting the fabrication procedure and chemical structure of the electroconductive mat by means of concurrent electrospraying/electrospinning process. **b** SEM images (inset: high-magnification view) of the electroconductive mat. **c** Cross-sectional SEM image of the electroconductive mat. **d** Photograph of large-scale electroconductive mat (= 10 × 10 cm^2).

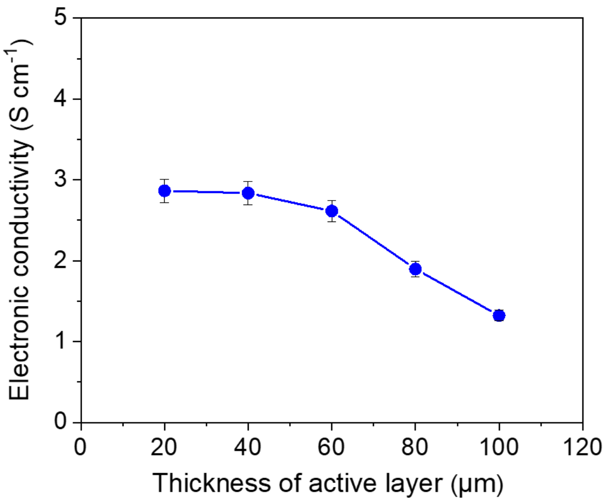

Supplementary Figure 4 | Electronic conductivity of the unit BNQS electrodes varying the thickness of active layer between electroconductive mat. The standard error was within 5%.

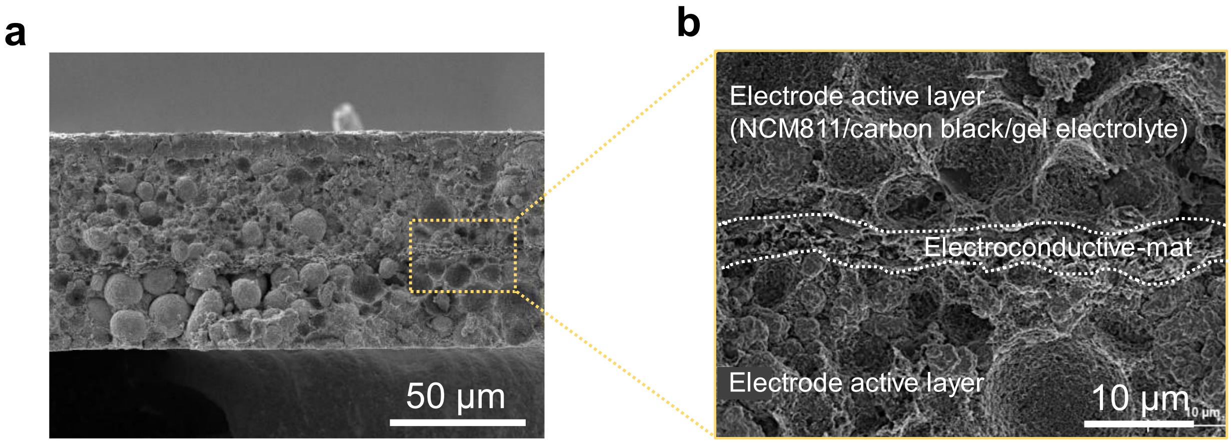

Supplementary Figure 5 | a, b Cross-sectional SEM images of the metal-current-collector-free BNQS electrode showing the intimate interfacial contact between the electroconductive mat and electrode active layer.

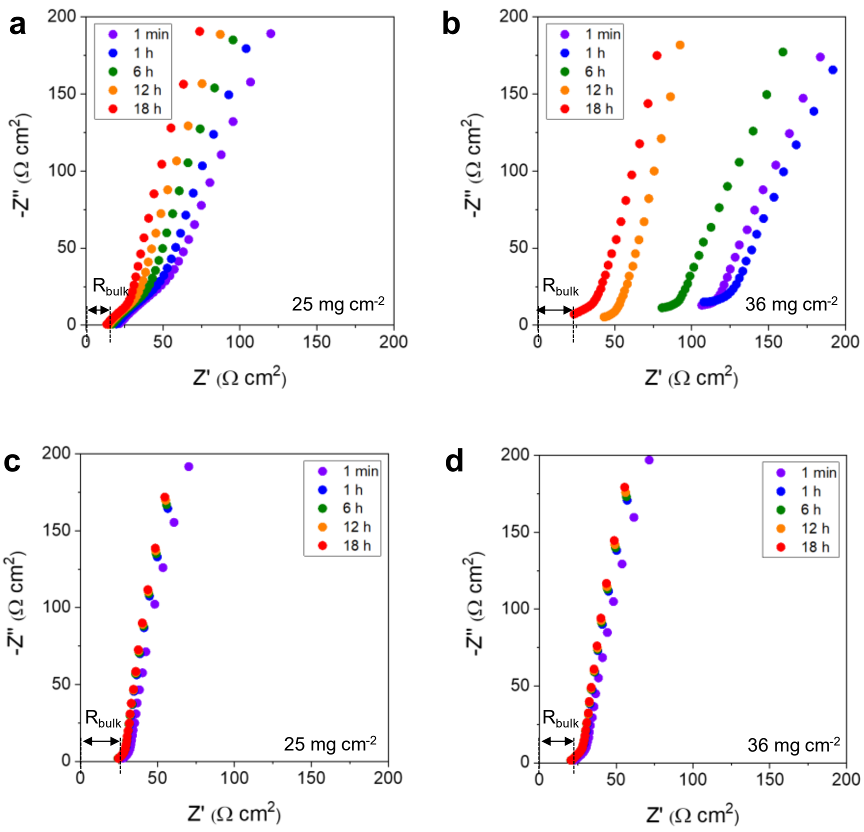

Supplementary Figure 6 | EIS analysis of the slurry-cast electrodes obtained by symmetric blocking cells (electrode|separator|electrode). Nyquist plots of the slurry-cast electrodes: **a** Areal-mass-loading = 25 mg cm^{-2}. **b** Areal-mass-loading = 36 mg cm^{-2}. Nyquist plots of the BNQS electrodes: **c** Areal-mass-loading = 25 mg cm^{-2}. **d** Areal-mass-loading = 36 mg cm^{-2}. The EIS measurement was conducted at 25°C.

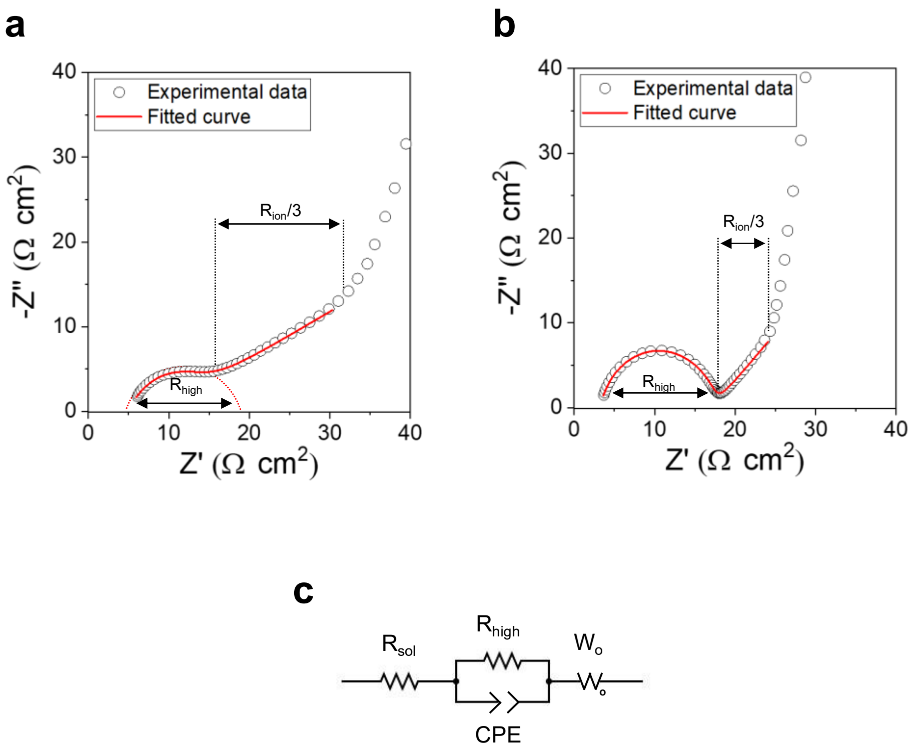

Supplementary Figure 7 | Nyquist plots of **a** slurry-cast and **b** BNQS electrodes obtained by a symmetric cell configuration with two identical electrodes at a state of charge (SOC) of 0 %, in which hollow symbols and solid lines represent experimental data and fitted curves based on a transmission line equivalent circuit model (TLM), respectively. The EIS measurement conducted at 25°C. **c** Transmission line equivalent circuit model (TLM).[1,2]

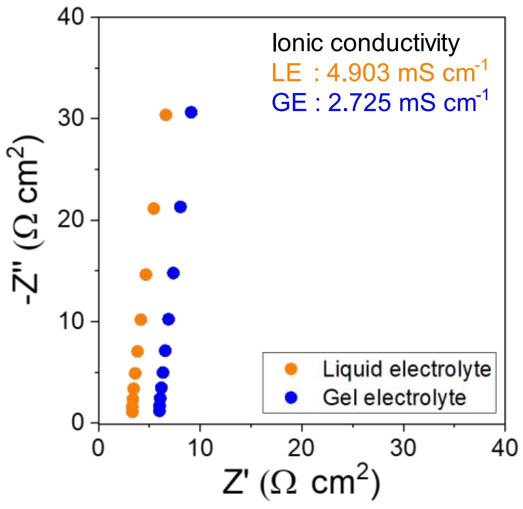

Supplementary Figure 8 | Ionic conductivities of the liquid electrolyte (1 M $LiPF_6$ in EC/PC, shown in orange) and gel electrolyte (liquid electrolyte/ETPTA monomer = 85/15 (w/w), shown in blue). The EIS measurement was conducted using a symmetric cell (SUS|separator|SUS) at 25°C.

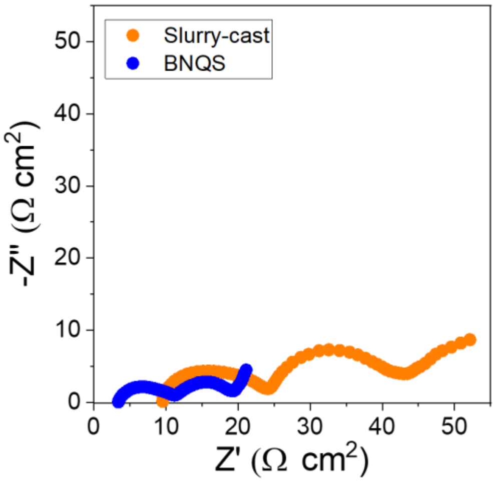

Supplementary Figure 9 | Nyquist plots (slurry-cast cathode versus BNQS cathode) of the Li metal cells (cathode||Li metal) at 100% SOC, in which the areal-mass-loading of cathodes was 36 mg cm^{-2}. The EIS measurement was conducted at 25°C.

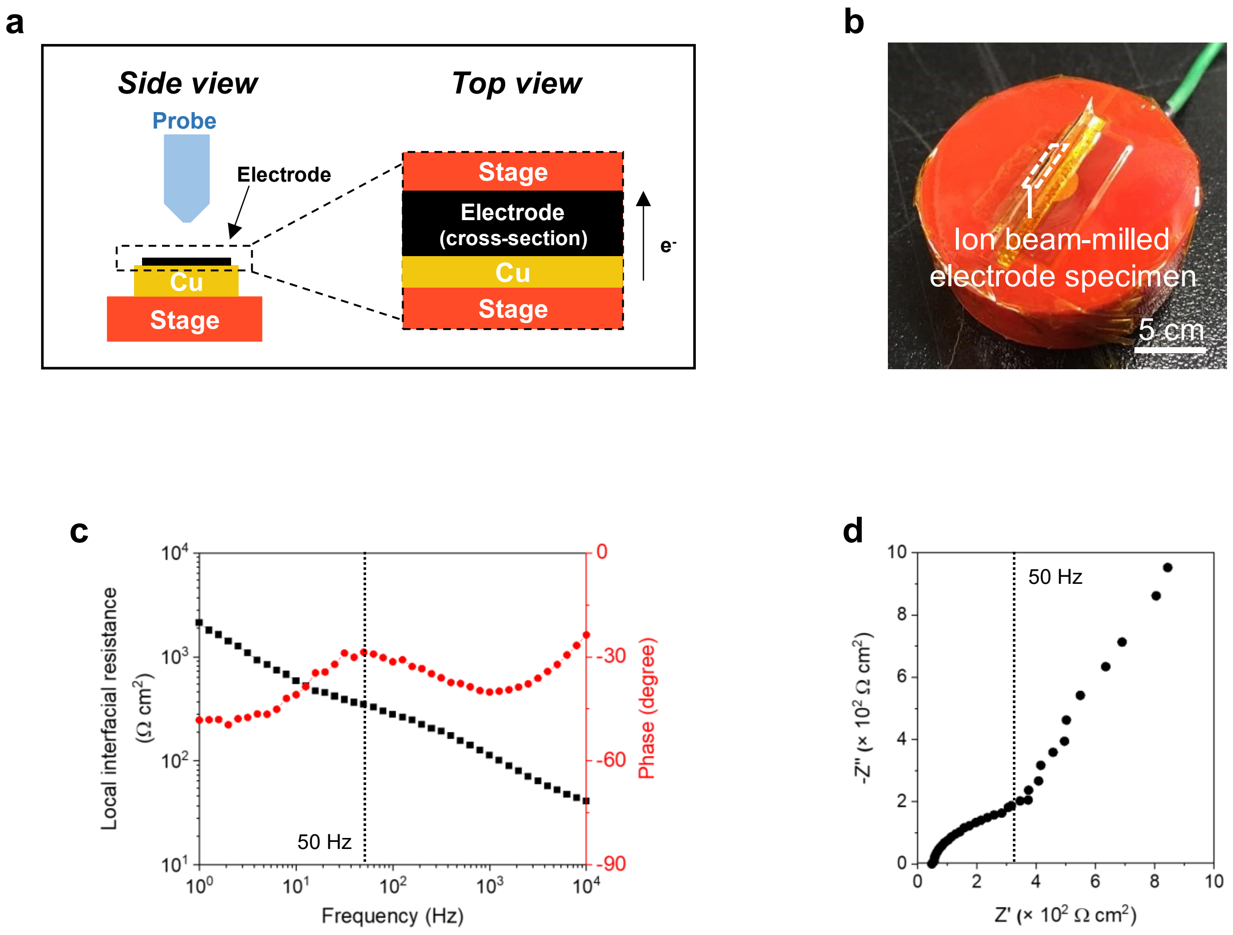

Supplementary Figure 10 | Description of the localized electrochemical impedance spectroscopy (LEIS) analysis. a Schematic illustration of the measurement. **b** Photograph of the electrode specimen. **c** LEIS frequency sweep of the BNQS electrode, in which the dotted line shows the characteristic frequency of charge transfer resistance. **d** Nyquist plot of the BNQS electrode. The LEIS area scan was conducted using an M470 scanning probe workstation (Biologic) at a fixed frequency of 50 Hz and an applied amplitude of 100 mV with 10-μm spacing.[3] This measurement was conducted at 25°C.To investigate the directional distribution of localized charge transfer resistance ($R_{l,ct}$), ion-milled electrodes were insulated except for the joint between the current collector and working electrode. The percent deviation of $R_{l,ct}$ was determined by the equation

$$Percent\ deviation\ of\ R_{l,ct} = \frac{R_{l,ct} - \overline{R_{l,ct}}}{\overline{R_{l,ct}}} \times 100$$

where $R_{l,ct}$ is an experimental value, and $\overline{R_{l,ct}}$ is an averaged value at the current collector surface (BNQS electrode = 333.1 Ω cm^2 and slurry-cast electrode = 2037 Ω cm^2).

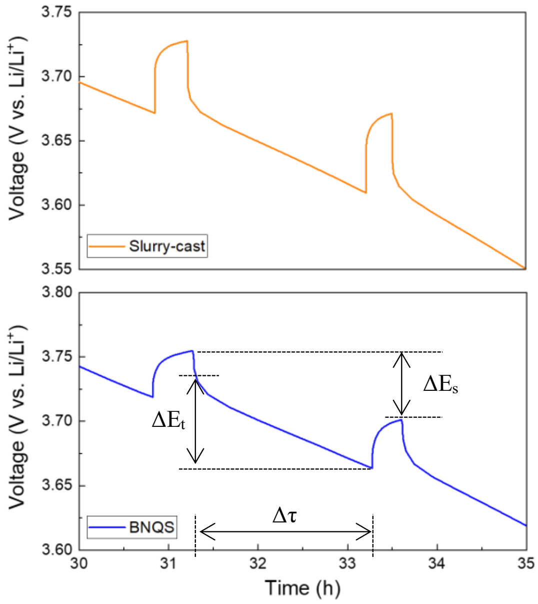

Supplementary Figure 11 | GITT profiles showing the discharging step of the cathodes around a nominal voltage of 3.74 V.

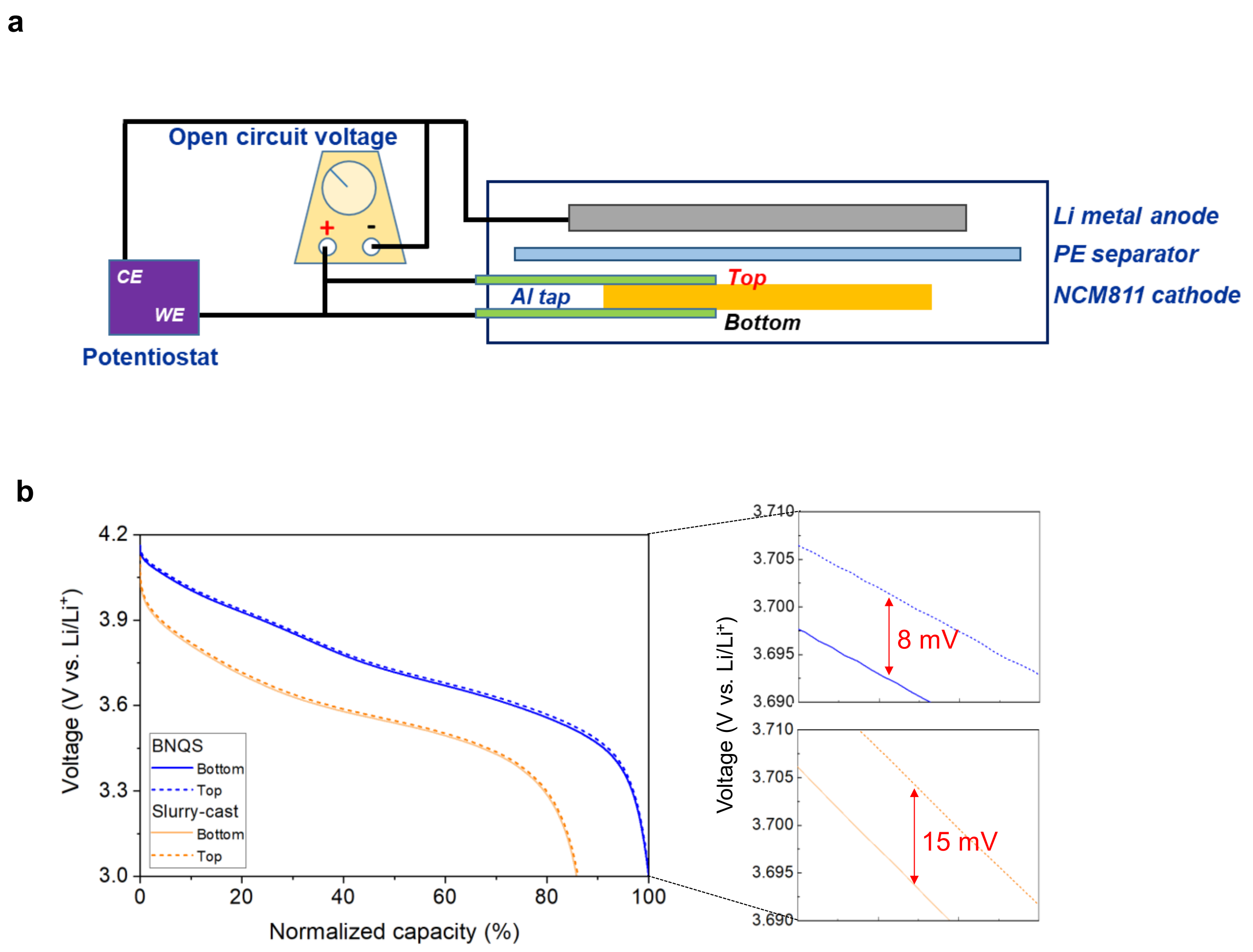

Supplementary Figure 12 | Overpotential distribution (*i.e.*, voltage difference between the top and bottom side) of the electrodes in through-thickness direction using an *in situ* measurement of electrode potential. a Schematic representation depicting a pouch cell designed for the *in situ* measurement of the overpotential distribution. **b** Voltage difference between the top (dotted line) and bottom (solid line) side of the cathodes at current density of 0.1 C/0.1 C (= 0.7 mA cm^{-2}).

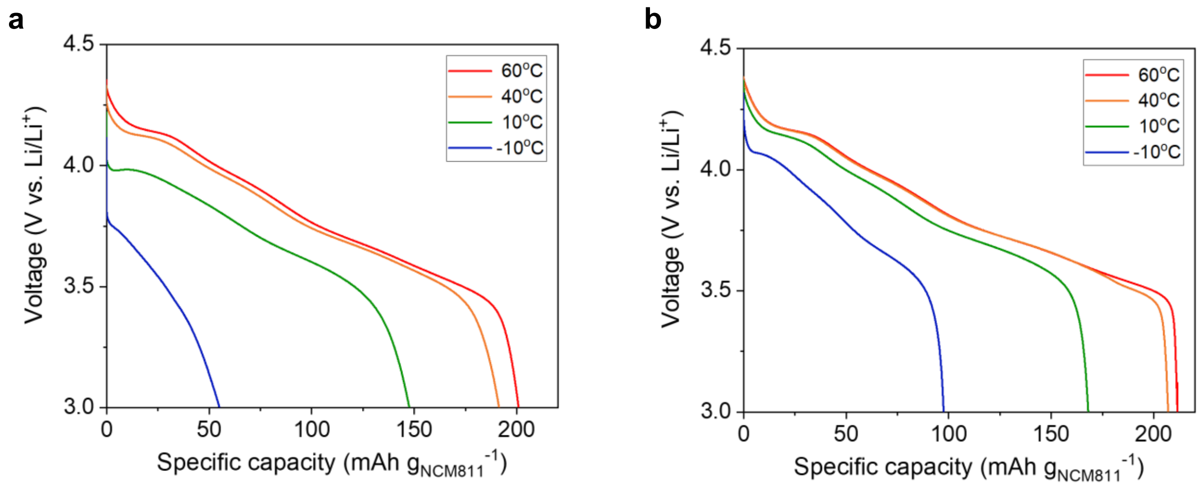

Supplementary Figure 13 | Discharge profiles of **a** slurry-cast and **b** BNQS cathodes (areal-mass-loading = 35 mg cm^{-2}) at various operating temperatures (varying from -10 to 60°C) under fixed discharge current density of 0.1 C (= 0.7 mA cm^{-2}).

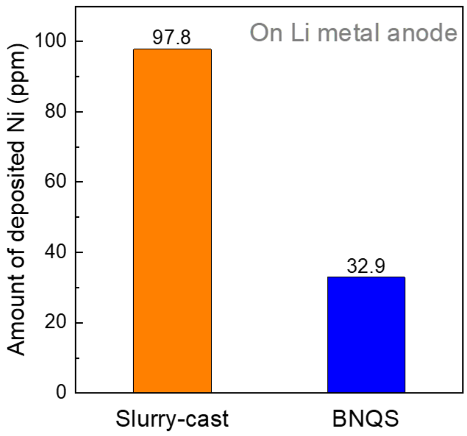

Supplementary Figure 14 | Amount of metallic Ni deposited on the Li metal anodes, which was measured using ICP-MS.

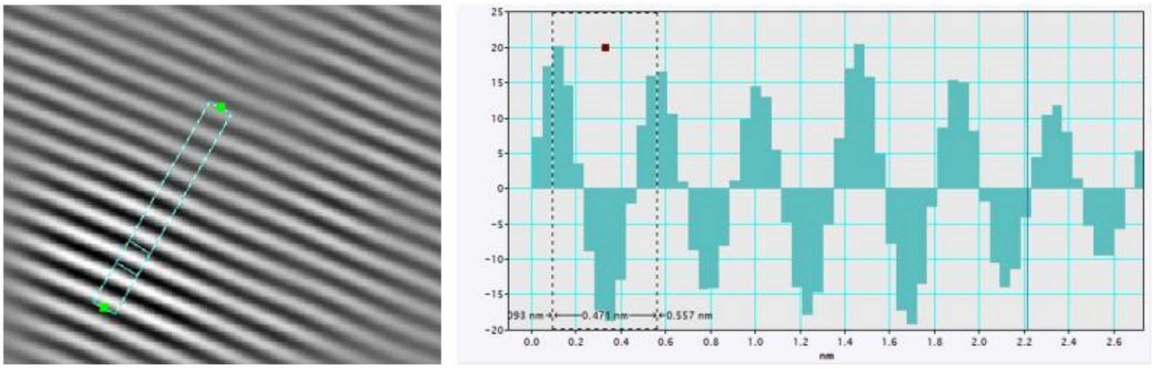

Supplementary Figure 15 | Inverse FFT and corresponding line profiles of the cycled NCM811 particles in the BNQS cathode.

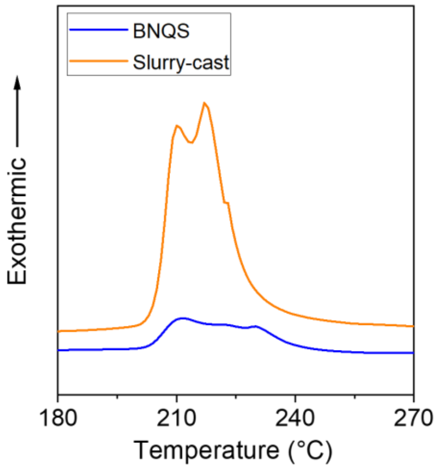

Supplementary Figure 16 | DSC thermograms of the 4.2 V-charged BNQS cathode (versus slurry-cast cathode) showing the interfacial exothermic reaction between the NCM811 and electrolytes.

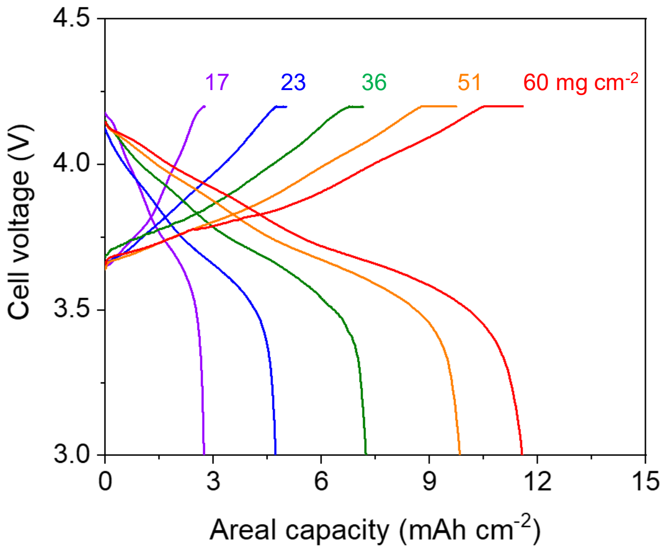

Supplementary Figure 17 | Charge/discharge profiles of the cells as a function of areal-mass-loading of the BNQS cathodes at charge/discharge current rate of 0.1 C/0.1 C (e.g., 1.1 mA cm^{-2} for 60 mg cm^{-2}) and voltage range of 3.0 – 4.2 V.

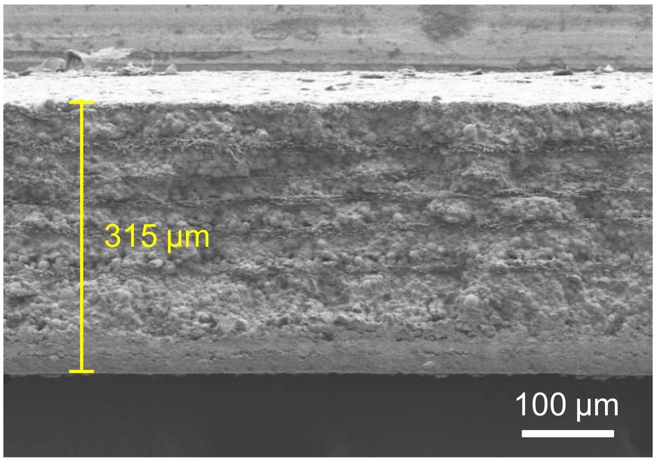

Supplementary Figure 18 | Cross-sectional SEM image of the high-mass-loading BNQS electrode electrode (thickness of 315 μm) with an areal-mass-loading of 60 mg cm^{-2}.

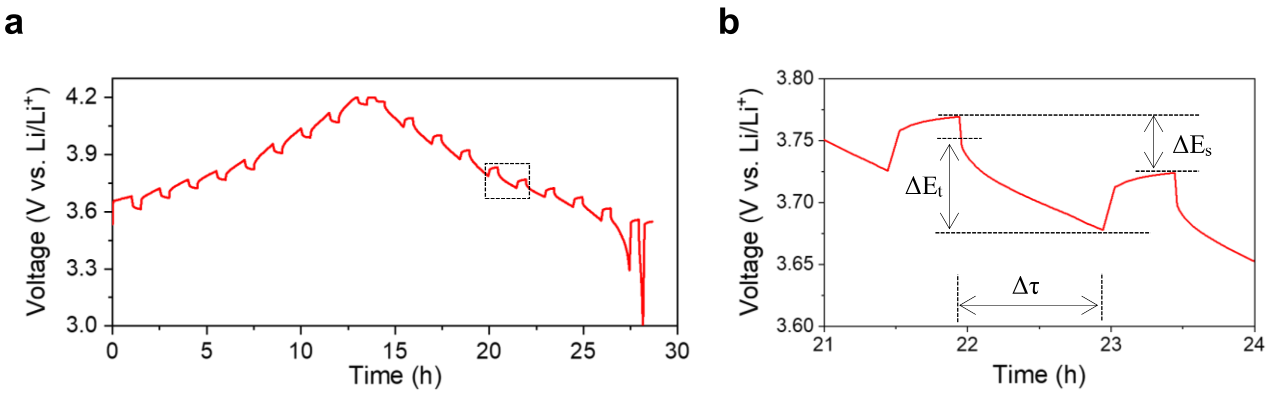

Supplementary Figure 19 | **a** GITT profiles of Li metal cells (BNQS cathode||Li metal anode) with an areal-mass-loading of 60 mg cm^{-2} upon repeated current stimuli at a charge/discharge current density of 0.1 C/0.1 C (= 1.16 mA cm^{-2}). **b** GITT profiles showing the discharging step of the cells around a nominal voltage of 3.74 V.

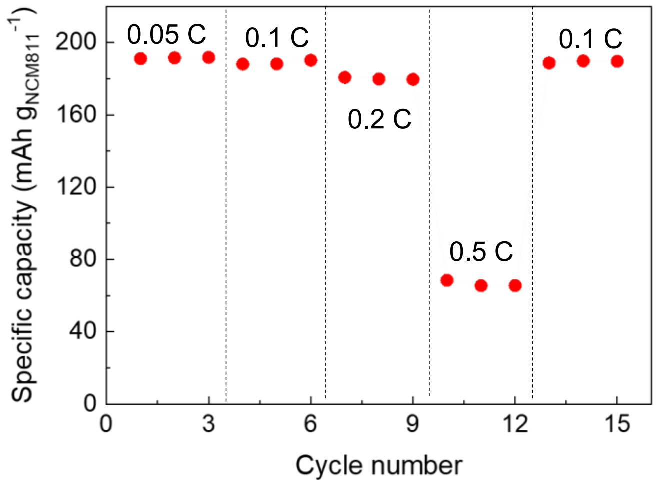

Supplementary Figure 20 | Discharge capacities Li metal cells (BNQS cathode||Li metal anode) with an areal-mass-loading of 60 mg cm^{-2} under varied discharge current densities (0.05 C (= 0.58 mA cm^{-2}) – 0.5 C (= 5.80 mA cm^{-2})) at a fixed charge rate of 0.05 C and a voltage range of 3.0 – 4.4 V.

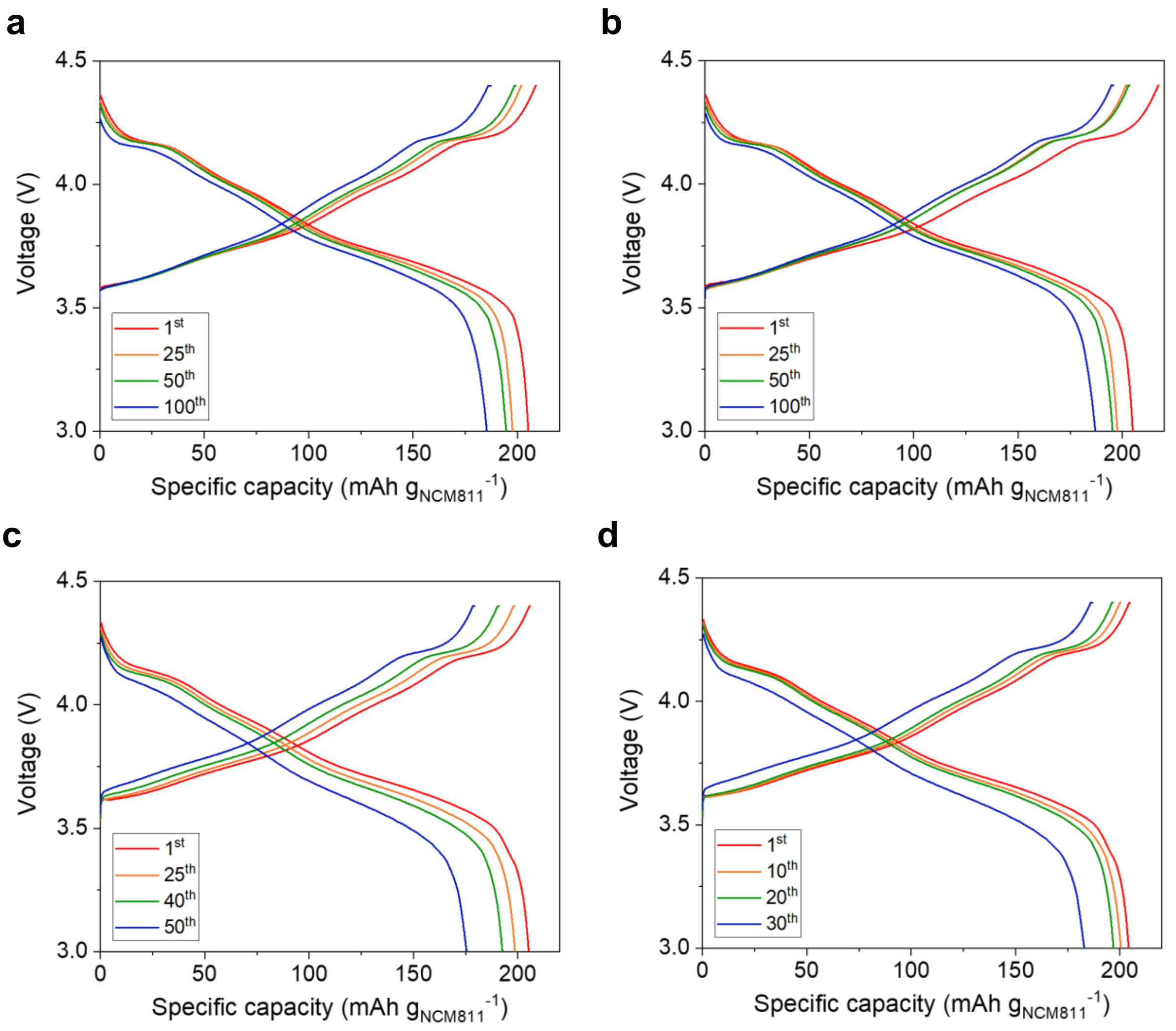

Supplementary Figure 21 | Charge/discharge profiles of the cell as a function of areal-mass-loading of the BNQS cathodes at charge/discharge current rate of 0.05 C/0.1 C and voltage range of 3.0 – 4.4 V: areal mass loading of **a** 17 mg cm^{-2} (1 C = 3.5 mA cm^{-2}), **b** 23 mg cm^{-2} (1 C = 4.7 mA cm^{-2}), **c** 51 mg cm^{-2} (1 C = 10.5 mA cm^{-2}), and **d** 60 mg cm^{-2} (1 C = 12.3 mA cm^{-2}).

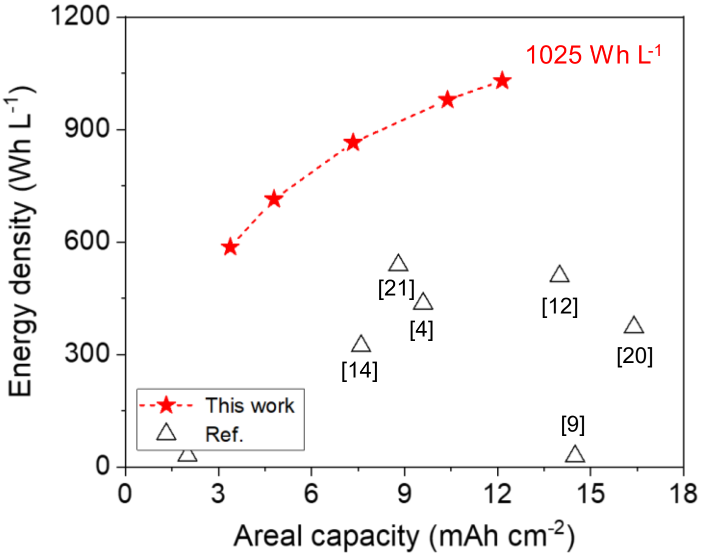

Supplementary Figure 22 | Energy densities of cells as a function of areal capacity (BNQS cathodes versus previously reported cathodes), in which the cell volume was estimated by considering those of cathodes, anodes, and separators.

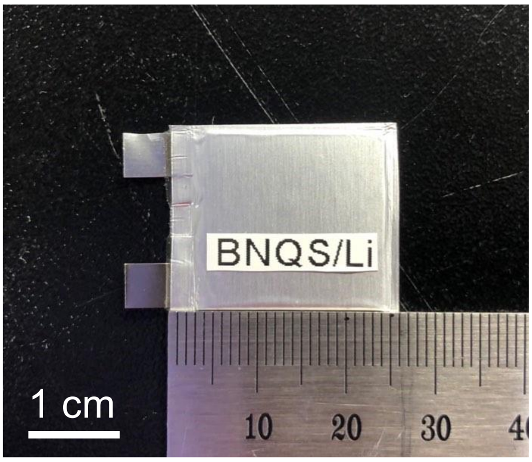

Supplementary Figure 23 | Photograph of the single-side pouch-type cell (26 × 24 mm^2 in size) composed of BNQS cathode (areal capacity of 12.1 mAh cm^{-2})||Li metal anode (areal capacity of 20 mAh cm^{-2}).

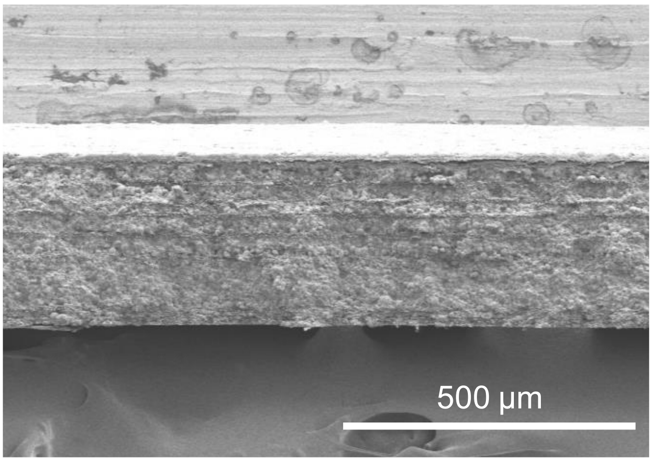

Supplementary Figure 24 | Cross-sectional SEM image of the BNQS cathode (areal-mass-loading of 60 mg cm^{-2}) after the cycling test at charge/discharge current rate of 0.05 C/0.1 C (= 0.6 mA cm^{-2}/1.2 mA cm^{-2}) (shown in Figure 6d,e). The cell (fully discharged to 3.0 V) was disassembled for this measurement.

Supplementary Table 1 | Basic information of the electroconductive-mat interlayers inserted in the BNQS electrode as a function of areal-mass-loading.

Areal-mass-loading of active materials ($mg\ cm^{-2}$)	Electroconductive-mat interlayers		
	Number	Areal-weight ($mg\ cm^{-2}$)	Thickness (μm)
16	1	0.8	9
23	2	1.2	18
36	3	1.6	27
51	6	2.8	54
60	7	3.2	63

Supplementary Table 2 | Comparison of the E/C (= electrolyte mass/cell capacity, g Ah^{-1}) ratio in the cells (BNQS electrode in this study versus previously reported high-mass-loading electrodes).

Concept	Electrolyte<br>g Ah^{-1}	Ref.
Quasi-solid-state	2.3	This work
Slurry-casting	-	4
	-	5
Magnetic alignment	-	6
SPS templating	-	7
3D printing	-	8
	-	9
	-	10
Ice templating	-	11
	-	12
Wood templating	13.8	13
	-	14
Conductive textile	-	15
	-	16
	-	17
Conductive percolation	-	18
	-	19
	-	20
	17.6	21

Supplementary Table 3 | The equation terms for the calculation of Li ion diffusion coefficients (D_{Li+}) of the cathodes. The diffusion coefficients were calculated based on the equation

$$D_{Li+} = \frac{4}{\pi \Delta \tau}\left(\frac{m_B V_M}{M_B S}\right)\left(\frac{\Delta E_s}{\Delta E_\tau}\right)^2$$

where m_B is assigned to the mass of the electrode active material, S is the geometric area of the electrode, M_B is the molar mass of the electrode material, V_M is the molar volume of the electrode material, and other parameters ($\Delta\tau$, ΔE_τ, and ΔE_S) in the equation are displayed in the GITT profiles shown in Supplementary Figure 11.

At 3.74 V	$\Delta\tau$	M_B	V_M	$m_B S^{-1}$	ΔE_s	ΔE_t	D_{Li+}
	(s)	(g mol^{-1})	(cm^3 mol^{-1})	(g cm^{-2})	(V)	(V)	(cm^2 s^{-1})
slurry-cast	7200	97.28	20.53	0.036	0.047	0.065	5.45 E-09
BNQS	7200	97.28	20.53	0.038	0.065	0.055	1.57 E-08

Supplementary Table 4 | The exothermic temperature peaks and exothermic heats from the DSC results regarding Supplementary Figure 16.

	1st T_{Peak} (°C)	2nd T_{Peak} (°C)	$\Delta H_{Exothermic}$ (J g^{-1})
slurry-cast	209.9	217.3	945.8
BNQS	211.6	229.9	219.3

Supplementary Table 5 | The equation terms for the calculation of Li ion diffusion coefficients (D_{Li+}) of the cathode. The diffusion coefficients were calculated based on the equation

$$D_{Li+} = \frac{4}{\pi\Delta\tau}\left(\frac{m_B V_M}{M_B S}\right)\left(\frac{\Delta E_s}{\Delta E_\tau}\right)^2$$

where m_B is assigned to the mass of the electrode active material, S is the geometric area of the electrode, M_B is the molar mass of the electrode material, V_M is the molar volume of the electrode material, and other parameters ($\Delta\tau$, ΔE_τ, and ΔE_S) in the equation are displayed in the GITT profiles shown in Supplementary Figure 19.

At 3.74 V	$\Delta\tau$ (s)	M_B (g mol^{-1})	V_M (cm^3 mol^{-1})	m_B S^{-1} (g cm^{-2})	ΔE_s (V)	ΔE_t (V)	D_{Li+} (cm^2 s^{-1})
	3600	97.28	20.53	0.060	0.040	0.074	1.63 E-08

Supplementary Table 6 | Comparison of major physical/electrochemical properties between the BNQS cathode (this study) and previously reported high-mass-loading cathodes. *For more details on specific energy and energy densities, see **Supplementary Table 7**.

Concept	Thickness	Loading of active materials	Areal capacity (Cathode sheet-based)	Theoretical capacity of active materials	Experimental capacity of active materials	Utilization	Specific energy* (Cell components)	Energy density*	Ref.
	μm	mg cm^{-2}	mAh cm^{-2}	mAh g_{AM}^{-1}	mAh g_{AM}^{-1}	(exp. capacity/ theor. capacity = %)	Wh kg^{-1}	Wh L^{-1}	
Quasi-solid-state	315	60	12.3 (0.1 C)	205 (NCM811 (4.4 V))	205	100	404 (cathode, anode, separator, electrolyte)	1025	This work
Slurry-casting	305	82	9.2 (0.1 C)	155 (NCM111)	112	72	206 (cathode, anode, separator, electrolyte (theoretically estimated))	436	4
	154	38	6.1 (0.1 C)	170 (NCM622)	158	93	246 (cathode, anode, separator, electrolyte (theoretically estimated))	-	5
Magnetic alignment	200	63	8.1 (0.05 C)	145 (LCO)	128	88	-	-	6
SPS templating	1000	140	21.3 (0.05 C)	170 (LFP)	144	85	-	-	7
3D printing	960	10	2.0 (1 C)	170 (LFP)	150	88	-	31	8
	1000	108	14.5 (0.013 C)	170 (LFP)	133	78	-	29	9
	1500	50	7.5 (-)	170 (LFP)	140	82	-	-	10
Ice templating	-	72	9.4 (0.2 C)	150 (LFMP)	130	86	550 (cathode)	-	11
	900	100	14.0 (0.1 C)	145 (LCO)	142	97	435 (cathode, anode, separator)	509	12

Wood templating	1000	169	21.9 (0.1 C)	145 (LCO)	106	73	219 (cathode, anode, separator, electrolyte)	566	13
	800	60	7.6 (0.05 C)	170 (LFP)	126	74	-	323	14
Conductive textile	600	145	22.9 (-)	170 (LFP)	158	93	-	-	15
	300	213	28 (0.025 C)	145 (LCO)	132	91	299 (cathode, anode)	-	16
	1200	89	10 (0.05 C)	155 (NCM111)	112	72	205 (cathode, anode, separator, electrolyte)	-	17
Conductive percolation	800	155	29.5 (0.06 C)	195 (NCM811 (4.3 V))	185	95	401 (cathode, anode, separator, electrolyte (theoretically estimated))	1030	18
	450	80	13.0 (0.1 C)	250 (OLO)	162	65	-	-	19
	480	108	16.4 (0.1 C)	170 (LFP)	152	89	-	373	20
	480	60	8.8 (0.049 C)	170 (LFP)	146	85	-	538	21

Supplementary Table 7 | Calculation details for the specific energies/energy densities of cells containing the BNQS cathodes.

As shown in Figure 6c, the specific energy of the Li metal cell is plotted. The equation[18] be derived according to,

[Eq.1] Specific energy (Wh kg^{-1})

$$=\frac{\text{Energy}}{\text{Mass of cell}}=\frac{\frac{\text{Energy}}{\text{Area}}}{\frac{\text{Mass of cell}}{\text{Area}}}=\frac{\text{Nominal Volatge} \times \text{C/A}}{\text{M}_{\text{cathode}}/\text{A}+\text{M}_{\text{anode}}/\text{A}+\text{M}_{\text{separator}}/\text{A}+\text{M}_{\text{electrolyte}}/\text{A}}$$

where $M_{cathode}$, M_{anode}, $M_{separator}$ and $M_{electrolyte}$ are the mass of cathode (including the electroconductive-mat interlayers and gel electrolyte), anode (consisting of Li metal (100 μm corresponding to an areal capacity of 20 mAh cm^{-2}) and Cu current collector (9 μm)), separator and injected electrolyte. C and A indicates capacity and area, respectively.

For the cell with charge cut-off voltage of 4.4 V, electrolyte in the cell was controlled as a E/C ratio of 2.3 g Ah^{-1} (= 1.1 g Ah^{-1} of gel electrolyte in the cathode and 1.2 g Ah^{-1} of injected electrolyte to electrochemically activate the separator and Li metal anode).

C/A	$M_{cathode}$/A	M_{anode}/A	$M_{separator}$/A	$M_{electrolyte}$/A	M_{total}/A	Specific energy
(mAh cm^{-2})	(mg cm^{-2})	(mg cm^{-2})	(mg cm^{-2})	(mg cm^{-2})	(mg cm^{-2})	(Wh kg^{-1})
12.3	83.8	13.2	2.0	14.6	113.6	404

The energy density of the Li metal cell is calculated according to,

[Eq.2] Energy density (Wh L^{-1})

$$= \frac{\text{Energy}}{\text{Thickness of cell}} = \frac{\text{Nominal Volatge} \times \text{C/A}}{T_{cathode} + T_{anode} + T_{separator}}$$

where $T_{cathode}$, T_{anode}, $T_{separator}$ are the thickness of cathode, anode (consisting of Li metal (100 μm) and Cu current collector (9 μm)), separator, respectively.

For the cell with charge cut-off voltage of 4.4 V,

C/A	$T_{cathode}$	T_{anode}	$T_{separator}$	Energy density
(mAh cm^{-2})	(μm)	(μm)	(μm)	(Wh L^{-1})
12.3	315	109	20	1025

Supplementary Table 8 | Parameters of the pouch-type cell, in which the cell was composed of the BNQS cathode (BNQS cathode (areal capacity of 12.1 mAh cm^{-2})|separator|Li metal anode (areal capacity of 20 mAh cm^{-2}). The area of the BNQS cathode, Li metal anode and separator were 20 × 20 mm^2, 21 × 21 mm^2 and 23 × 23 mm^2, respectively. The specific energies and energy densities were estimated based on the experimentally measured weight and volume of the cell (including packaging substances).

Cell components	Mass (mg)	Areal mass (mg cm^{-2})	Areal capacity (mAh cm^{-2})	Thickness (μm)
BNQS cathode	330	83	12	314
Li-metal anode	64	13	20	109
Separator	7	1	-	16
Injected electrolyte	59	-	-	-
Pouch substances	106	-	-	150

Pouch-type cell parameters	Discharge state (Voltage = 3.0 V)
Mass (mg)	566
Thickness (μm)	589
Capacity (mAh)	48
Energy (mWh)	182
Specific energy (Wh kg^{-1})	321
Energy density (Wh L^{-1})	772

Supplementary reference list

1. Zhao, Z. *et al.* Sandwich, Vertical-Channeled Thick Electrodes with High Rate and Cycle Performance. *Adv. Funct. Mater.* **29**, 1809196 (2019).
2. Sun, H. *et al.* Three-dimensional holey-graphene/niobia composite architectures for ultrahigh-rate energy storage. *Science* **356**, 599-604 (2017).
3. Huang, V. M. *et al.* Local electrochemical impedance spectroscopy: A review and some recent developments. *Electrochim. Acta* **4**, 8048 (2011).
4. Singh, M., Kaiser, J. & Hahn, H. A systematic study of thick electrodes for high energy lithium ion batteries. *J. Electroanal. Chem.* **782**, 245-249 (2016).
5. Gallagher, K. G. *et al.* Optimizing Areal Capacities through Understanding the Limitations of Lithium-Ion Electrodes. *J. Electrochem. Soc.* **163**, A138-A149 (2015).
6. Sander, J. S., Erb, R. M., Li, L., Gurijala, A. & Chiang, Y. M. High-performance battery electrodes via magnetic templating. *Nat. Energy* **1**, 16099 (2016).
7. Elango, R., Demortière, A., De Andrade, V., Morcrette, M. & Seznec, V. Thick Binder-Free Electrodes for Li-Ion Battery Fabricated Using Templating Approach and Spark Plasma Sintering Reveals High Areal Capacity. *Adv. Energy Mater.* **8**, 1703031 (2018).
8. Wei, T. S., Ahn, B. Y., Grotto, J. & Lewis, J. A. 3D Printing of Customized Li-Ion Batteries with Thick Electrodes. *Adv. Mater.* **30**, 1703027 (2018).
9. Wang, J. *et al.* Toward High Areal Energy and Power Density Electrode for Li-Ion Batteries via Optimized 3D Printing Approach. *ACS Appl. Mater. Interfaces* **10**, 39794-39801 (2018).
10. Sun, K. *et al.* 3D printing of interdigitated Li-ion microbattery architectures. *Adv. Mater.* **25**, 4539-4543 (2013).
11. Zhao, Z. *et al.* Sandwich, Vertical-Channeled Thick Electrodes with High Rate and Cycle Performance. *Adv. Funct. Mater.* **29**, 1809196 (2019).
12. Huang, C. & Grant, P. S. Coral-like directional porosity lithium ion battery cathodes by ice templating. *J. Mater. Chem. A* **6**, 14689-14699 (2018).
13. Lu, L. L. *et al.* Wood-Inspired High-Performance Ultrathick Bulk Battery Electrodes. *Adv. Mater.* **30**, 1706745 (2018).
14. Chen, C. *et al.* Highly Conductive, Lightweight, Low-Tortuosity Carbon Frameworks as Ultrathick 3D Current Collectors. *Adv. Energy Mater.* **7**, 1700595 (2017).
15. Hu, L. *et al.* Lithium-Ion Textile Batteries with Large Areal Mass Loading. *Adv. Energy Mater.* **1**, 1012-1017 (2011).
16. Zheng, J. *et al.* Nonplanar Electrode Architectures for Ultrahigh Areal Capacity Batteries. *ACS*

Energy Lett. **4**, 271-275 (2018).

17 Wang, J. S., Liu, P., Sherman, E., Verbrugge, M. & Tataria, H. Formulation and characterization of ultra-thick electrodes for high energy lithium-ion batteries employing tailored metal foams. *J. Power Sources* **196**, 8714-8718 (2011).

18 Park, S.-H. *et al.* High areal capacity battery electrodes enabled by segregated nanotube networks. *Nat. Energy* **4**, 560-567 (2019).

19 Kim, J.-M., Park, C.-H., Wu, Q. & Lee, S.-Y. 1D Building Blocks-Intermingled Heteronanomats as a Platform Architecture For High-Performance Ultrahigh-Capacity Lithium-Ion Battery Cathodes. *Adv. Energy Mater.* **6**, 1501594 (2016).

20 Li, H. *et al.* Ultrahigh-Capacity and Fire-Resistant LiFePO4-Based Composite Cathodes for Advanced Lithium-Ion Batteries. *Adv. Energy Mater.* **9**, 1802930 (2019).

21 Kuang, Y. *et al.* Conductive Cellulose Nanofiber Enabled Thick Electrode for Compact and Flexible Energy Storage Devices. *Adv. Energy Mater.* **8**, 1802398 (2018).
